# Combinatorial extracellular matrix tissue chips for optimizing mesenchymal stromal cell microenvironment and manufacturing

**DOI:** 10.1038/s41536-025-00408-z

**Published:** 2025-04-22

**Authors:** Ishita Jain, Alex H. P. Chan, Guang Yang, Hao He, Johnny Lam, Kyung Sung, Ngan F. Huang

**Affiliations:** 1 Department of Cardiothoracic Surgery, Stanford University, Stanford, CA 94305 USA; 2 Stanford Cardiovascular Institute, Stanford University, Stanford, CA 94305 USA; 3 Epicrispr Biotechnologies, South San Francisco, CA USA; 4 Center for Biologics Evaluation and Research, Food and Drug Administration, Silver Spring, MD 20993 USA; 5 Center for Tissue Regeneration, Repair and Restoration & Geriatric Research, Education, and Clinical Center, Veterans Affairs Palo Alto Health Care System, Palo Alto, CA 94304 USA; 6 Department of Chemical Engineering, Stanford University, Stanford, CA 94305 USA

**Keywords:** Mesenchymal stem cells, Stem-cell research

## Abstract

Despite the therapeutic potential of mesenchymal stromal cells (MSC), there is limited understanding of optimal extracellular matrix (ECM) environments to manufacture these cells. We developed tissue chips to study the effects of multi-factorial ECM environments under manufacturable stiffness ranges and multi-component ECM compositions. Manufacturing qualities of cell expansion potential, immunomodulation, and differentiation capacity were examined. The results show stiffness effects, with 900 kPa substrates supporting higher proliferation and osteogenic differentiation, along with anti-inflammatory IL-10 expression, whereas 150 kPa substrates promoted adipogenic differentiation at 150 kPa, suggesting that optimal ECM environments may differ based on manufacturing goals. ECM biochemistries containing fibronectin and laminin further modulated MSC manufacturing qualities across various stiffnesses. Proteomic and transcriptomic analyses revealed unique ECM combinations that induced higher levels of angiogenic and immunomodulatory cytokines, compared to single factor ECMs. These findings demonstrate that optimized ECM environments enhance MSC manufacturing quality.

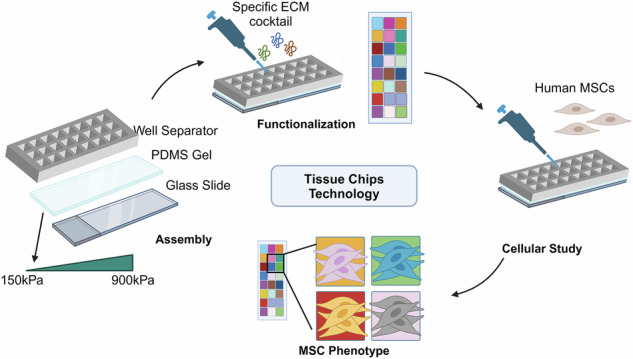

## Introduction

Mesenchymal stromal cells (MSCs), or commonly known as mesenchymal stem cells, reside in numerous tissues in the body, including the bone marrow and adipose tissue^1^. MSCs are capable of differentiating into a variety of cellular lineages, secreting therapeutic factors, and conferring immunomodulatory effects^2^. These properties make MSCs a candidate cell type for regenerative medicine applications^3^. Consequently, human MSCs have been studied in over 2000 clinical trials spanning a range of disease indications including cardiovascular disease, neurological diseases, and cancer^4^.

Despite the promising therapeutic potential of MSCs, efficient and scalable manufacturing processes remain a critical bottleneck for their widespread clinical application. Biochemically, the physiological environment of the bone marrow, adipose tissue, and other tissues in which MSCs reside is generally a milieu rich in extracellular matrix (ECM) proteins, including collagen (such as collagen types I, II, III, IV), fibronectin, laminin, proteoglycans, and glycosaminoglycans^5–8^. In the bone marrow, the MSCs reside in a perivascular niche as part of the stromal cell population^9^. This perivascular niche accounts for 90% of bone marrow volume^10^. Even with the high cellular density of this niche, it is rich in ECM components. Recapitulation of the perivascular niche using ECM cues for hematopoietic stem cells biology and function has been reported in literature^9,11^ but has not been explored well for MSCs. Conventional in vitro MSC manufacturing primarily relies on tissue culture polystyrene dishes, which lack the complex ECM signaling cues present in native tissues. Additionally, as MSC quality and behavior may vary among donors, the extracellular cues for optimal expansion and function may need to be tailored for each donor line. These factors contribute to variability in MSC proliferation, differentiation, and therapeutic functionality, posing challenges for clinical scalability. To address these challenges, there is an urgent need for advanced manufacturing platforms that can both replicate the native microenvironment of MSCs and allow for donor-specific customization.

There is limited understanding of how ECM microenvironmental cues can potentially benefit MSC manufacturing. Previous studies primarily focused on substrate stiffness effects on MSCs for proliferation, differentiation and paracrine signaling^12^, in which 22 kPa–100 kPa stiffness environments promoted proliferation and osteogenic differentiation, whereas 0.5 kPa substrates promoted adipogenic differentiation^13–15^. Other studies using specific substrate stiffnesses could support long-term culture of MSCs^16^. Despite underscoring the importance of mechanical factors on MSC behavior, these studies do not include complex ECM biochemical components in which MSCs reside^6–8,17,18^.

Accordingly, we custom engineered tissue chips spanning a range of stiffnesses that can be: (1) easily manufactured and robustly scalable (150 kPa–900 kPa), while still being notably lower in stiffness than conventional tissue culture polystyrene; (2) cost-effective and scalable to a similar degree as tissue culture polystyrene; and (3) can serve as a platform to optimize ECM environments for various MSC lines. These substrates were further immobilized with multi-component ECM protein combinations comprised of collagen II, collagen III, collagen IV, fibronectin, and laminin, as they are proteins commonly present within the bone marrow or other native MSC niches^5–8^. Manufacturing qualities including cell proliferation, differentiation, and immunomodulation capacity were examined in parallel, concomitantly with transcriptomic and proteomic profiling. Our results show that combinatorial ECMs differentially modulate cell proliferation, differentiation, and immunomodulation through stiffness dominant transcriptional signaling pathways. Nevertheless, specific ECM combinations were shown to override the effects of stiffness to direct the cellular phenotype of MSCs. We further demonstrate the clinical translation potential of tissue chips by benchmarking MSC quality against transcriptional datasets of freshly isolated human bone marrow cells containing MSCs. Together, these findings support the utility of tissue chip technology to optimize ECM environments to manufacture MSCs.

## Results

### 150 kPa stiffness and ECM combinations promote adipogenic differentiation potential

The tissue chip platform was composed of mechanically tunable polydimethylsiloxane (PDMS) substrates encompassing a range of stiffnesses that can be manufactured in a reproducible manner. The substrates were immobilized onto microscope slides (25 × 75 mm) and then surface functionalized by poly-D-dopamine for subsequent ECM deposition (Supplementary Fig. 1). To increase the throughput of the chip, a detachable 24-well chamber was secured onto the substrate using detachable clamps. To study the effects of combinatorial ECMs that better mimic the complex native ECM milieu, we selected five ECMs associated with bone marrow or adipose tissue in which MSCs reside, denoted as collagen II (2), collagen III (3), collagen IV (4), fibronectin (F) and laminin (L). Henceforth, the ECM combinations are denoted using the general scheme of E_1_E_2…_E_i_ for a combination composed of the ECMs E_1_ + E_2_…+E_i_. For example, the ECM combination 4 + F + L is denoted as 4FL. These ECMs were systematically deposited onto the substrates all possible (1-, 2-, 3-, 4-, and 5-factor) combinations, all at equimolar ratios to maintain the same total protein concentration. These resulting 31 ECM combinations were evaluated at 3 stiffnesses (150 kPa, 500 kPa and 900 kPa), leading to a total of 93 unique microenvironmental conditions.

**Fig. 1:**
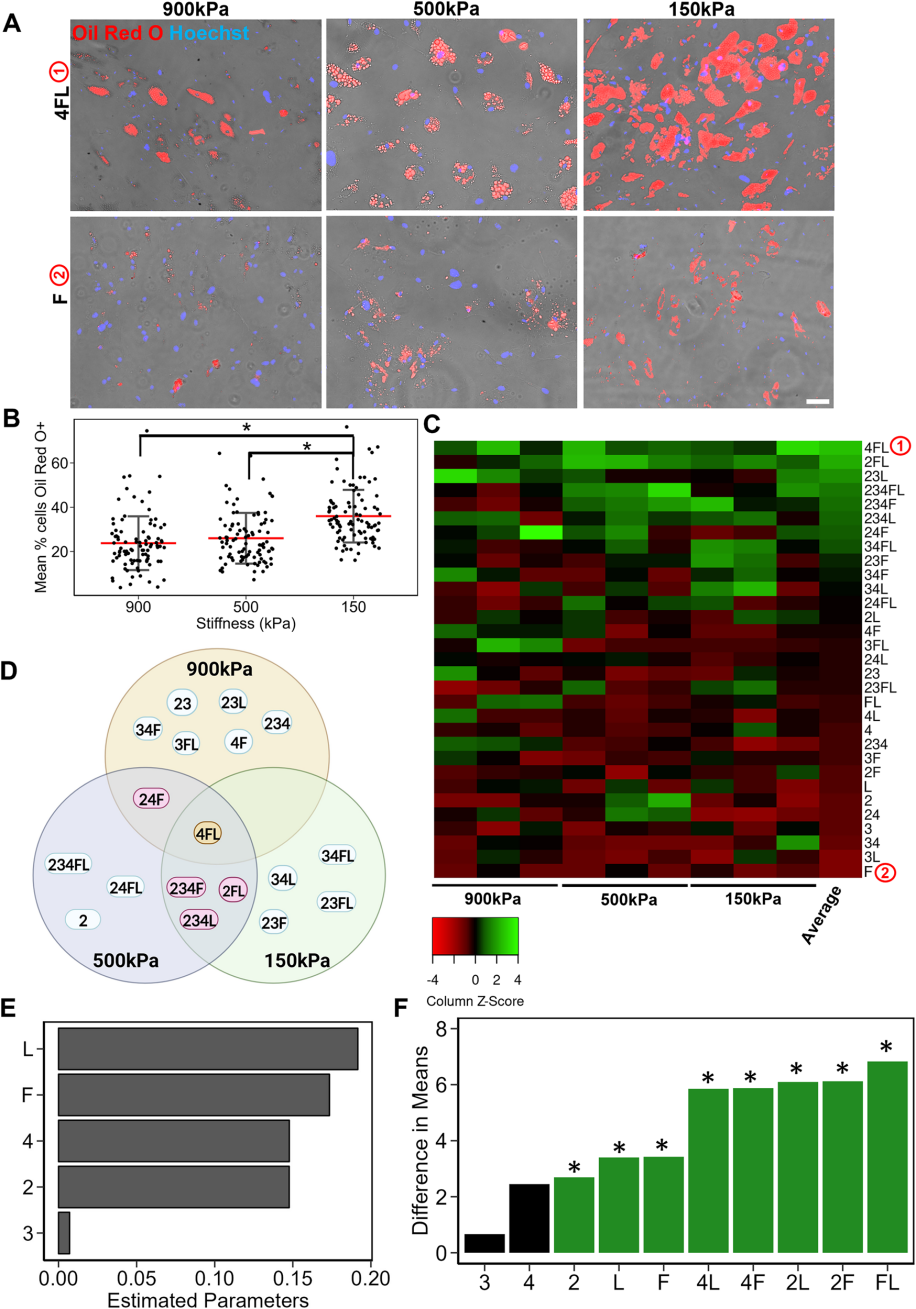
Differential effect of adipogenesis on tissue chips. **A** Representative overlayed fluorescent and brightfield images of MSCs that underwent adipogenic differentiation on the tissue chips. Red: Oil Red O; Blue: Hoechst. Scale Bar: 100 µm. **B** Dot plot of Oil Red O%+ MSCs as a function of stiffness. Each dot represents a single sample on a specific ECM, where the red line represents the mean value. **C** Heatmap of scaled Oil Red O%+ MSCs on different ECM combinations and stiffness. The ECMs are ranked from high to low based on their averages across stiffnesses. **D** Venn diagram of top ranked ECMs for adipogenesis for each stiffness, with common ECMs across two stiffnesses and three stiffnesses highlighted. **E** Bar plot of estimated coefficients in the linear regression model of adipogenesis as a function of individual ECM components, ranked from high to low. **F** Bar plot of the difference in average Oil Red O%+ MSCs when a certain ECM combination is present versus absent. Green denotes statistically significant combination (*P < 0.05). Data corresponds to at least three biological replicates.

Using this tissue chip platform, we first examined the effect of combinatorial ECM composition and stiffness after 21 days of culture in adipogenic induction media. The adipogenic phenotype of MSCs was quantified using an Oil Red O stain, which stains for lipid droplets, and expressed as the percentage of cells that stains positively for Oil Red O (Fig. 1A, B). Based on adipogenic differentiation, the differential effects of substrate stiffness and combinatorial ECMs were observed. With respect to stiffness effects alone, a significantly higher percentage of cells stained positively for Oil Red O at 150 kPa substrates (36 ± 12%), compared to both 900 kPa (24 ± 12%, *P* < 0.05) and 500 kPa (26 ± 11%, *P* < 0.05) (Fig. 1A, B). This is consistent with published literature showing that adipogenesis is favorable in low stiffness environments^13,14^.

The effects of combinatorial ECMs on adipogenesis were visualized in a heat map that ranked the ECM combinations across all stiffnesses, based on the average percent positive Oil Red O cells for each composition. ECM compositions showing the highest level of adipogenesis are ranked at the top of the heat map, whereas ECMs supporting to lowest adipogenesis were ranked at the bottom (Fig. 1C). Based on the heat map, the ECM compositions 4FL (depicted as ① on the heat map) and 2FL exhibited the highest Oil Red O%+ cells across all stiffness, whereas ECM compositions F (depicted as ② on the heat map) and 3L exhibited the average lowest Oil Red O%+ cells. Consistent with published findings from other cellular systems, many of the single-component ECMs generally appear in the lower parts the heat map, whereas combinatorial ECMs are ranked among the top ECM combinations^19–21^. However, when evaluated at each stiffness independently, the top ECM combinations varied to some degree. By visualizing the intersection of ECM combinations and stiffnesses in the form of a Venn diagram, we identified the ECMs that best promoted adipogenesis for each stiffness and discovered ECMs that intersect at multiple stiffnesses. For example, the tri-component combination of 4FL was the only common ECM combination among all stiffness. Additionally, the top adipogenesis-inducing ECMs on 900 kPa appeared to have the least degree of intersection with the 500 kPa or 150 kPa stiffnesses (Fig. 1D). This data demonstrates that both stiffness and ECM combinations modulated adipogenesis.

To further analyze the data, we employed a linear regression model, which is frequently used to distill the effects of multi-factorial parameters in quantifiable coefficient estimates. This approach leads to the identification of the significant contributors and compare the effect of each factor with the others, as was demonstrated previously^21–24^. Using the data derived from full-factorial combinations of ECMs, we applied a linear regression model to elucidate the main effects of ECM components on Oil Red O expression. The estimated parameter, which is derived from the regression model, is a measure of influence of the ECM on Oil Red O%, in which the more positive the value is associated with higher influence. The estimated parameter, as a function of the five ECM components (2, 3, 4, F, L), suggests that L had the overall highest positive main effect on adipogenesis (Fig. 1E). We additionally performed higher order generalized linear model (GLM) analysis, which quantifies the effect of the presence of a specific ECM protein within all ECM compositions, compared to its absence among all ECM compositions, as was done previously^25,26^. This analysis reveals the main and interactional effect of the five ECM factors. This analysis showed that the presence of 2, F, and L within a combination significantly led to a positive regulation of adipogenesis. Statistically significant relationships were also found among the two-factor combinations of 4L, 4F, 2F, 2L, and FL, which had an even higher positive regulation on adipogenesis than the single-factor ECMs (Fig. 1F). This analysis was consistent with the rank ordered ECM combinations, which showed the combinations containing FL were among the highest ranked combinations (Fig. 1B). Overall, all two factor combinations of 2, F, L (with the exception of 24) had a significantly positive effect on the Oil Red O%+ MSCs. Together, these results demonstrate that 150 kPa substrates and most two-factor combinations of 2, F, L supported the highest levels of adipogenesis in MSC manufacturing.

### 900 kPa Stiffness and ECMs Containing Fibronectin Enhance Osteogenic Differentiation Potential of MSCs

**Fig. 2:**
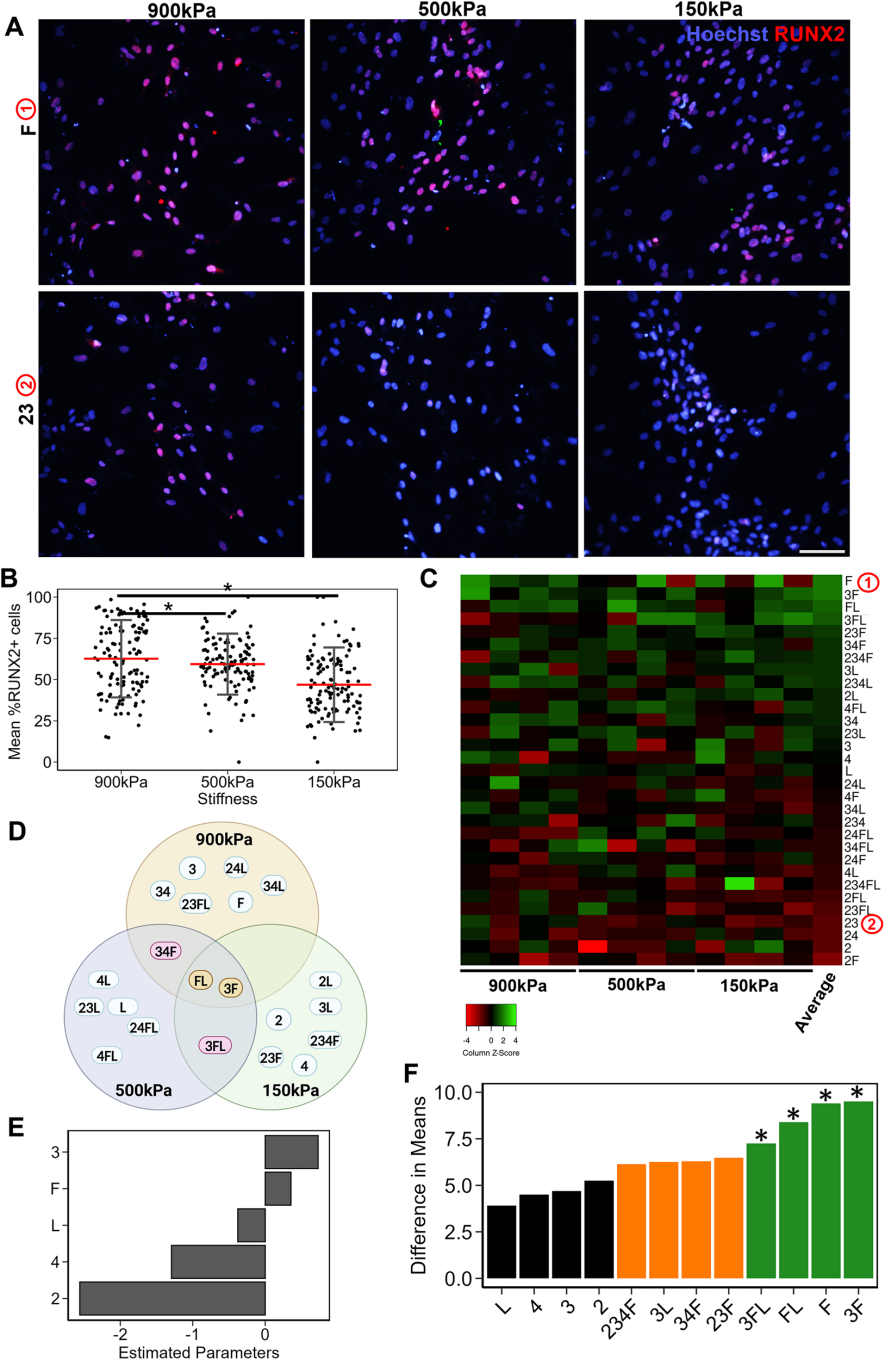
Osteogenic differentiation of MSCs on tissue chips. **A** Representative overlayed images depicting RUNX2 expression in MSCs that underwent osteogenic differentiation on the tissue chips. Red: RUNX2; Blue: Hoechst. Scale Bar: 100 µm. **B** Dot plot of RUNX2%+ MSCs as a function of stiffness. Each dot represents a metric for a single image on a specific ECM, and the red line represents mean. **C** Heatmap of scaled RUNX2%+ MSCs on different ECM combinations and stiffness. The ECMs are ranked from highest to lowest based on their averages across stiffnesses. **D** Venn diagram of top ECMs for osteogenesis for each stiffness, with common ECMs across two stiffnesses and three stiffnesses highlighted. **E** Bar plot of estimated coefficients in the linear regression model of osteogenesis as a function of individual ECM components, ranked from high to low. **F** Bar plot of difference in average RUNX2%+ MSCs when a certain ECM combination is present versus absent. Green: Significant combination (*P* < 0.05); Orange: Close to Significant (*P* < 0.1). Data presented here corresponds to at least three biological replicates. * denotes *P* < 0.05.

Since the optimal ECMs may differ depending on the differentiation lineage, we further investigated ECM effects on osteogenesis. MSCs were cultured in osteogenic differentiation media for 21 days in the tissue chips and then immunofluorescently stained for RUNX2, a prominent osteogenic differentiation transcription factor. On 900 kPa substrates, the cells showed a significantly higher percentage (63 ± 23%, *P* < 0.05) of nuclear-localized RUNX2, compared to that on 500 kPa (59 ± 18%, *P* < 0.05) or 150 kPa (46 ± 22%) substrates (Fig. 2A, B). The stiffness effects concur with findings from other groups showing improved osteogenic differentiation on higher stiffness substrates^13–15^.

Notably, ECM effects were observed in the heatmap (Fig. 2C) that ranked the ECMs from high to low RUNX2%+ cells across all ECMs and three stiffnesses. The ECM composition of F (as depicted as ① the heatmap) and 3F showed the highest percentage of RUNX2+ cells. ECMs compositions 23 (depicted as ② on the heatmap) and 24 were among the lowest ranked for the RUNX2%+ cells. Interestingly, the top two ECM compositions favoring osteogenesis (F and 3F, Fig. 2C) were among the lower ranking ECMs for adipogenic differentiation (Fig. 1C). However, there were also multiple ECM combinations in common among the top 10 ECMs for both osteogenic and adipogenic differentiation, such as 23F, 34F, 234F and 234L. This finding suggests that some ECM compositions are suitable for single lineage differentiation, whereas others are compatible for multi-lineage differentiation. We further compared the top 9 ECMs that resulted in the highest RUNX2%+ cells for the three stiffnesses (Fig. 2D). It is shown that ECM combinations FL and 3F were amongst the common top ECM across all stiffness. Compared to adipogenesis, there were less common top ECM combinations between stiffnesses, pointing towards a stiffness dominant effect on osteogenic differentiation.

To quantify these stiffness and ECMs effects, we analyzed the full-factorial dataset of three stiffness and 31 ECM combinations using linear regression and GLM models. Linear regression revealed that 2, 4, and L generally had a negative effect on osteogenic differentiation, whereas F and 3 positively modulated osteogenic differentiation (Fig. 2E). Furthermore, GLM modeling was performed to analyze factor effects and interactions of the different ECM combinations. The combination of 3FL, FL, F and 3F were among the most significant in promoting RUNX2+ expression (Fig. 2F). Overall, these results demonstrate that 900 kPa stiffness and the ECM compositions containing F promote the osteogenic differentiation of MSCs.

### 900 kPa stiffness and ECMs containing fibronectin and laminin support MSC immunomodulation

**Fig. 3:**
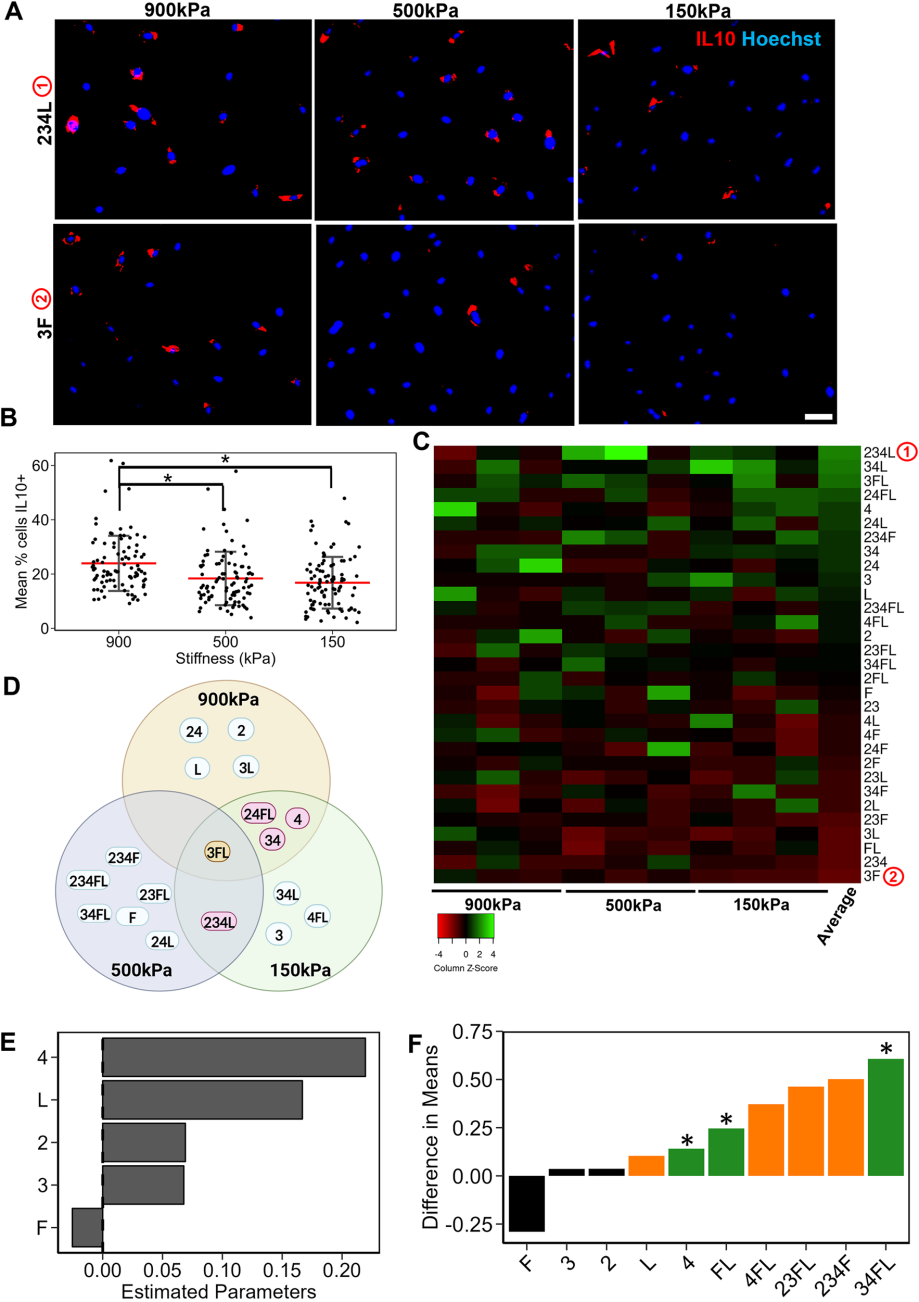
Immunomodulation of MSCs on tissue chips. **A** Representative images of depicting IL-10 expression in MSCs that underwent immunostimulation using IFNγ. Red: IL-10; Blue: Hoechst33342 for cell nuclei. Scale Bar: 100 µm. **B** Dot plot of IL-10%+ MSCs as a function of stiffness. Each dot represents a metric for a single image on a specific ECM, red line represents mean. **C** Heatmap of scaled IL-10%+ MSCs on different ECM combinations and stiffness. The ECMs are ranked highest to lowest based on their averages across stiffnesses. **D** Venn diagram of top ECMs for immunomodulation for each stiffness, with common ECMs across two stiffnesses and three stiffnesses highlighted. **E** Bar plot of estimated coefficients in the linear regression model of immunomodulation as a function of individual ECM components, ranked high to low. **F** Bar plot of difference in average IL-10%+ MSCs when a certain ECM combination is present versus absent. Green: Significant combination (*P* < 0.05) Orange: Close to significant (*P* < 0.1). Data presented here corresponds to at least three biological replicates. * denotes *P* < 0.05.

An important property of MSCs that is favorable for cell therapy is their immunomodulation capacity. Therefore, we next assessed the effect of substrate stiffness and combinatorial ECMs on their immunomodulatory response to exogeneous interferon gamma (IFN-γ), a pro-inflammatory cytokine associated with many disease etiologies^27–29^. The immunomodulatory effect of MSCs was quantified by the expression of IL-10, an anti-inflammatory cytokine^29^. The quantification of cells expressing IL-10 (Fig. 3A, B) shows a higher percentage on 900 kPa substrates (24 ± 10%), compared to both 500 kPa (18 ± 10%, *P* < 0.05) and 150 kPa substrates (17 ± 10%, *P* < 0.05).

Furthermore, combinatorial ECMs also modulated IL-10 expression, as depicted by the average IL-10%+ cells for each ECM combination across all stiffnesses (Fig. 3C). It was observed that the ECM combination 234L, (depicted as ① on the heat map) and 34L exhibited the average highest IL-10%+ cells across all stiffness. In contrast, the ECM combinations 234 and 3F, (depicted as ② on the heat map) exhibited the average lowest IL-10%+ cells (Fig. 3C). Full-factorial analysis also revealed examples of non-intuitive effects of the ECMs combinations. For example, the combination of FL showed synergistic effects on immunomodulation, compared to the single factors of F and L alone (Fig. 3C). On the other hand, the combination of 4L showed an inhibitory effect, compared to 4 and L alone. These examples highlight non-intuitive cellular responses to complex ECM cues. We next compared the top 8 ECM combinations that maximized the %IL-10 expression at each stiffness to examine the commonality of ECM effects across the stiffness range (Fig. 3D). The Venn diagram highlight the finding that most top-ranked ECM compositions at each stiffness were combinatorial ones. Some of the top-ranked ECMs showed intersections with more than one stiffness, but 3FL was the only ECM that intersected among all three stiffnesses. Together, this data suggested that both stiffness and ECM compositions modulated IL-10 expression in MSCs.

With the availability of full-factorial combinations of ECMs, we applied a linear regression model to reveal the main effects of ECM components on MSC immunomodulation capacity. Estimated parameters analysis revealed that 4 had the overall highest positive effect on immunomodulation, whereas the presence of F within an ECM combination led to a significant negative effect (Fig. 3E). Higher order GLM analysis further revealed a significant positive effect of multi-factorial ECM such as 34FL and FL on IL-10 expression (Fig. 3F). Additionally, we showed that F alone had a strong negative effect on immunomodulation, but the combination of FL had a significant positive effect on IL-10 expression (Fig. 3F), which is a non-intuitive cellular response that can be uniquely revealed by full-factorial experimental design. These models demonstrate that 900 kPa substrates and ECMs containing FL enhanced the manufacturing of immunomodulatory MSCs.

### MSC expansion is stiffness-dominated with additive effect of combinatorial ECMs

**Fig. 4:**
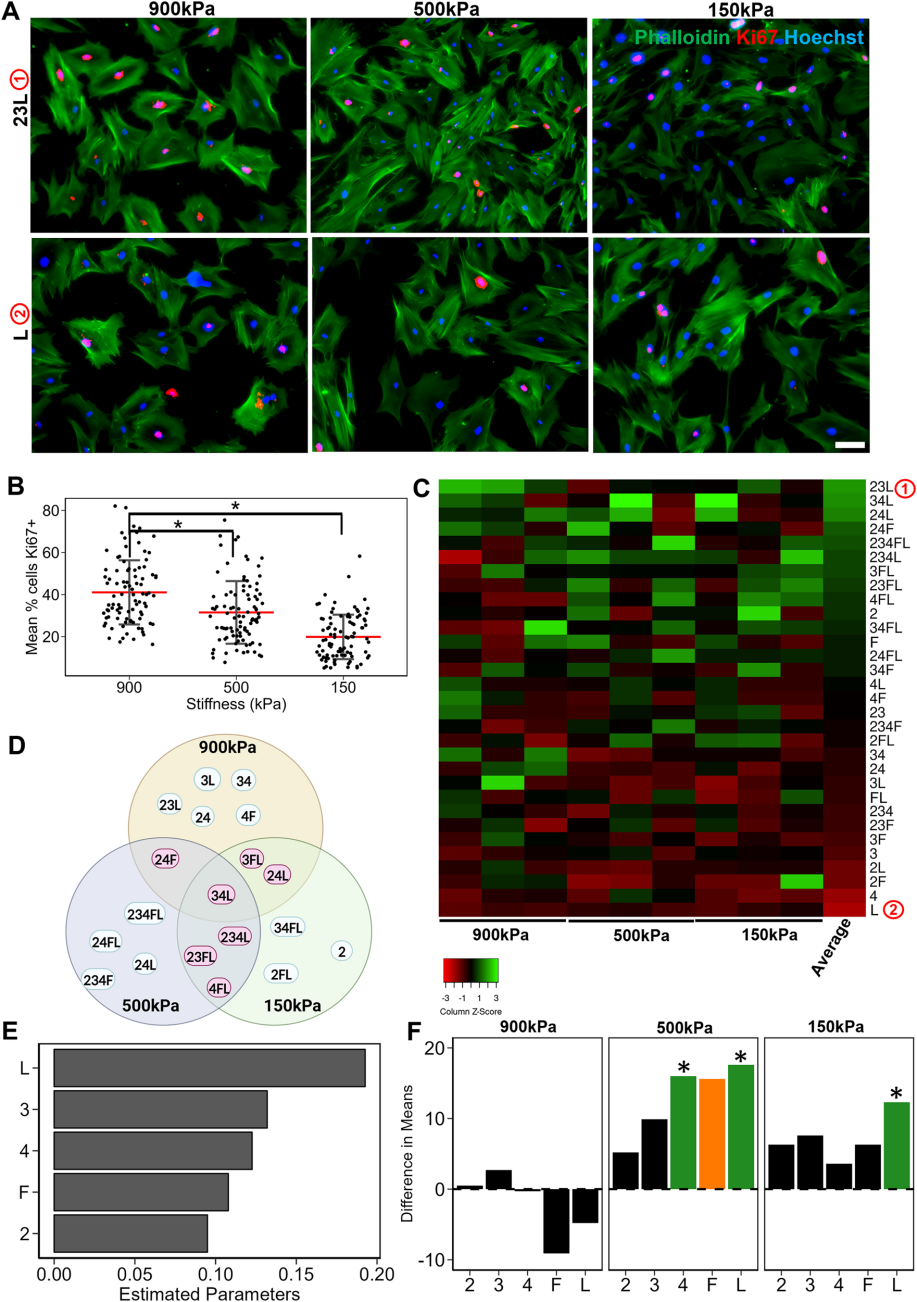
Proliferation of MSCs on tissue chips. **A** Representative images of Ki67 expression in MSCs cultured on tissue chips for 48 h Red: Ki67; Blue: Hoechst33342 for cell nuclei; Green: Phalloidin staining of F-actin. Scale Bar: 100 µm. **B** Dot plot of Ki67%+ MSCs at each stiffness. Each dot represents a metric for a single image on a specific ECM. Red line represents mean. **C** Heatmap of scaled Ki67%+ cells on different ECM combinations and stiffness. The ECMs are ranked high to low based on their averages across stiffnesses. **D** Venn diagram of top ECMs for proliferation for each stiffness, with common ECMs across two stiffnesses and three stiffnesses highlighted. **E** Bar plot of estimated coefficients in the linear regression model of proliferation as a function of individual ECM components, ranked high to low. **F** Bar plot of difference in average Ki67%+ MSCs when a certain ECM combination is present versus absent. Green: Significant combination (*P* < 0.05) Orange: Close to significant (*P* < 0.1). Data presented here corresponds to at least three biological replicates. * denotes *P* < 0.05.

As a measure of in vitro manufacturing potential, we further quantified the expansion of MSCs in combinatorial microenvironments using Ki67, an S-phase cell cycle marker. Quantification of Ki67 revealed a significantly higher Ki67%+ cells on 900 kPa (41 ± 15%), compared to 500 kPa (32 ± 15%, *P* < 0.05) and 150 kPa (20 ± 11%, *P* < 0.05) substrates. (Fig. 4A, B). Furthermore, notable ECM compositional differences were observed, where the three-factor ECM combination of 23L led to a higher percentage of Ki67 expression, compared to L alone (Fig. 4A). This result is consistent with the heat map depicting the ranked average Ki67%+ cells for each ECM combination across all stiffnesses (Fig. 4C). The heat map showed that the ECM combination 23L (depicted as ① on the heat map) and 34L exhibited the highest Ki67%+ cells across all stiffness, whereas L (depicted as ② on the heat map) exhibited the average lowest Ki67%+ cells (Fig. 4C). We then visualized the top ECM combinations across three stiffnesses and showed that the majority of the top ECMs were combinatorial ECMs (Fig. 4D), consistent with the findings derived from differentiation and immunomodulatory studies (Figs. 1D–3D). Additionally, there was a high degree of intersection between the top-ranked ECMs between the 150 kPa and 500 kPa substrates. To confirm these findings, MSC proliferation was quantified by direct cell counting on representative ECMs that either supported (i.e., 234L) or inhibited (2F) Ki67 expression on 500 kPa substrates. Consistent with Ki67 staining, MSCs on 234L led to a 44% population increase of MSCs after 24 h, whereas MSC culture on 2F resulted in a negligible growth rate.

Next, using linear regression models, we show that L had the overall highest positive effect on Ki67 expression, based on estimated parameters (Fig. 4E). Higher order GLM analysis revealed a significant positive effect on the presence of 4 or L on 500 kPa only (Fig. 4F). On 150 kPa substrates, L had the most significant positive effect on proliferation, whereas there was no significant effect of ECM on 900 kPa (Fig. 4F). This result, combined with the highest proliferation on 900 kPa, suggests a stiffness dominated effected on 900 kPa. Together, this data demonstrates stiffness-dependent ECM effects on proliferation, where the underlying stiffness dominated the overall proliferative phenotype and that the effect was additive in the presence of certain ECMs.

### Stiffness and combinatorial ECM effects on angiogenic and immunomodulatory cytokines in MSCs

**Fig. 5:**
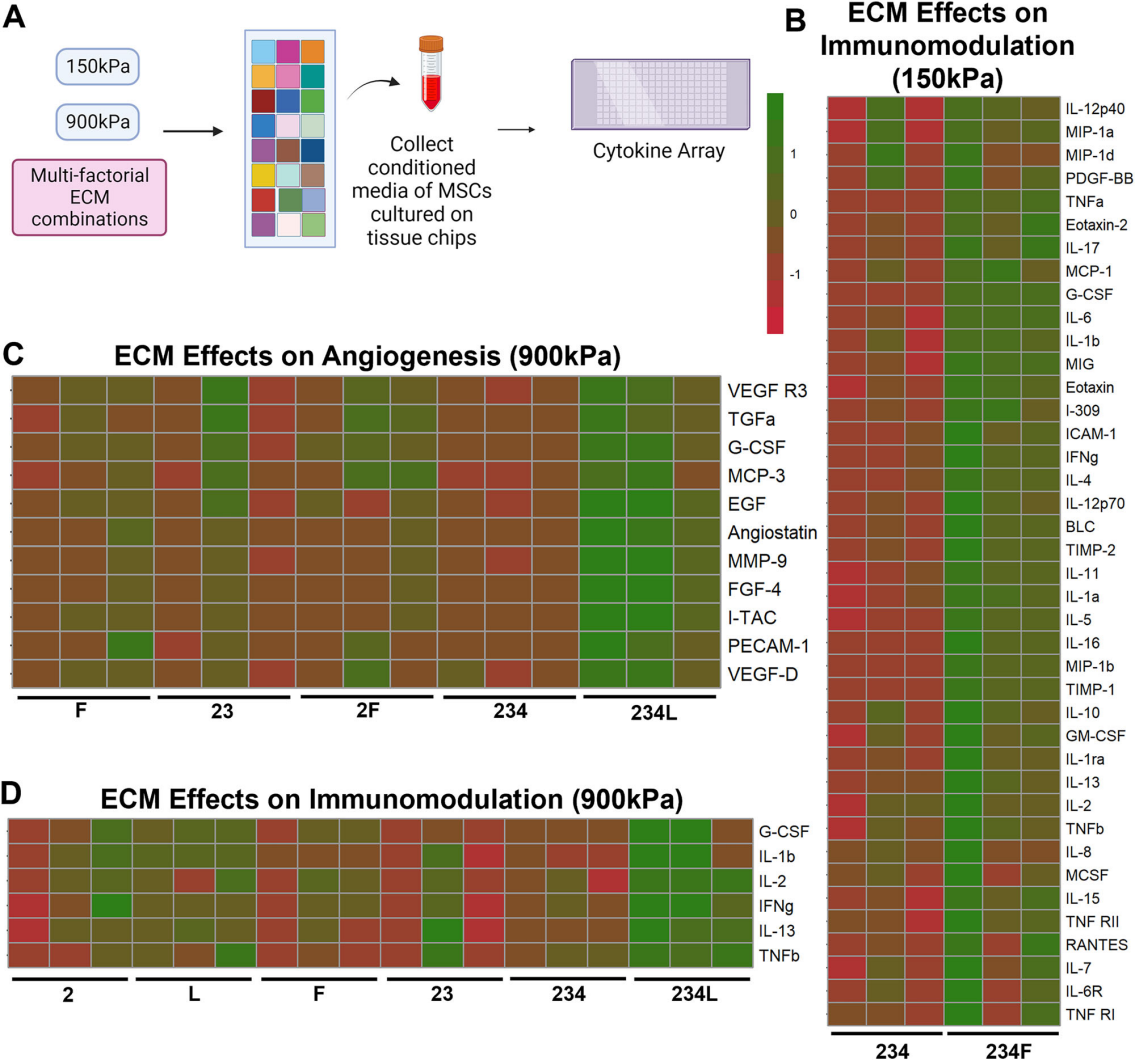
Proteomic analysis of angiogenic and immunomodulatory cytokines. **A** Schematic experimental design of proteomics study where MSCs were cultured on a subset of ECM conditions and then conditioned media was collected after 48 h. **B** Heatmap of scaled quantities of angiogenic cytokines secreted by MSCs on 900 kPa as a function of different combinatorial ECMs. **C** Heatmap of scaled quantities of immunomodulation cytokines secreted by MSCs on 900 kPa as a function different combinatorial ECMs. **D** Heatmap of scaled quantities of immunomodulation cytokines secreted by MSCs on 150 kPa as function of different combinatorial ECMs. Data presented here corresponds to at least three biological replicates. Created in BioRender. Huang, N. (2025) https://BioRender.com/l04s048.

To reveal the molecular pathways by which combinatorial ECMs influence MSC phenotype and function, proteomic analysis of secreted cytokines from conditioned media for a subset of ECMs at two different stiffnesses was performed (Fig. 5A). We examined cytokines associated with immunomodulation or angiogenesis, as they are important properties of MSC for various cell therapy applications. Proteomic assessment revealed that the ECM combination 234L significantly increased the levels of angiogenic and immunomodulatory cytokines, compared to two-factor and single-factor ECMs on 900 kPa substrates. In particular, vascular endothelial growth factor-D (VEGF-D), VEGF receptor-3 (VEGFR3), and matrix metalloproteinase-9 (MMP-9) were among the angiogenic cytokines that were significantly enhanced, resulting in a two-fold increase on 234L, compared to F, 23, 2F and 234 on 900 kPa (*P* < 0.05) (Fig. 5C). Additionally, interleukin-2 (IL-2) and IL-13, which are cytokines that inhibit inflammation by regulating T cells, along with tumor necrosis factor-b (TNFb) that plays a role in tumor immunity, were among the immunomodulation cytokines that were increased on 234L, compared to 2, L, F, 23 and 234 on 900 kPa (*P* < 0.05) (Fig. 5D). In comparison, on the 150 kPa substrates, a significant increase in immunomodulatory markers was observed on 234F, compared to 234 (Fig. 5B). Together, these data show that the four-factor ECM combinations promoted significantly more enhancement in angiogenic and immunomodulatory cytokine release, compared to lower order combination counterparts.

### Tissue chips reveal substrate stiffness and combinatorial ECM effects on MSC transcriptional signature

**Fig. 6:**
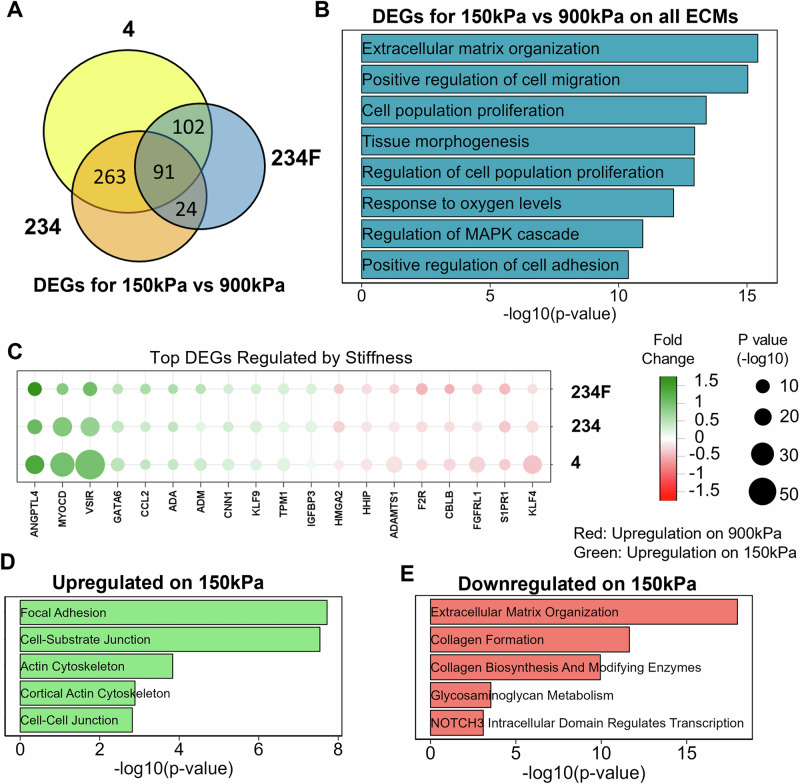
Transcriptional analysis of stiffness effects on MSCs. **A** Venn Diagram of Differentially Expressed Genes (DEGs) for MSCs cultured on 150 kPa vs 900 kPa substrates on ECMs compositions 4, 234 and 234F. **B** Gene enrichment bar plot for top genesets that were enriched in the 91 commons DEGs for 150 kPa vs 900 kPa for all ECMs. **C** Dot plot of expression of the top DEGs (x-axis) from 91 common DEGs for 150 kPa vs 900 kPa substrates on the different ECMs (y-axis). Red: Upregulation on 900 kPa; Green: Upregulation on 150 kPa. **D** Gene enrichment bar plot of overall DEGs upregulated on 150 kPa, independent of the ECM. **E** Gene enrichment bar plot of overall DEGs downregulated on 150 kPa, independent of the ECM.

To complement proteomic profiling, we further employed RNA Sequencing to reveal the effects of combinatorial ECMs, when compared to their single-factor components. In particular, transcriptional profiling was carried out to compare the effects of 4 to that of combinatorial ECMs 234 and 234F at both 150 kPa and 900 kPa stiffnesses. For each pair-wise comparison on specific ECM compositions, the number of differentially expressed genes (DEGs) between 150 kPa and 900 kPa were denoted in a Venn Diagram to highlight the similarities and differences across the ECMs (Fig. 6A). There were statistically significant DEGs that were specific to an ECM combination, with a fraction in common between two ECM combinations and a total of 91 DEGs in common between all three ECM combinations (P_adj_<0.05). The pathway and process enrichment analysis on those 91 genes reveals a pronounced enrichment for ECM organization and cell proliferation-related pathways among the DEGs across all ECM combinations (Fig. 6B). Furthermore, among the top DEGs between 150 kPa and 900 kPa across all ECMs (Fig. 6C). *MYOCD* (muscle cell marker), *VSIR* (immunoregulatory receptor) and *GATA6* (differentiation marker during embryogenesis) were significantly upregulated on 150 kPa; whereas genes like *KLF4* (cellular proliferation marker), *S1PR1* (vascular development marker) and *FGFRL1* (stem cell viability and proliferation) were upregulated on 900 kPa. To confirm these findings, we performed quantitative PCR and found similar fold changes in gene expression of *MYOCD* and *ANGPTL4*, when comparing the expression of MSCs cultured on 150 kPa vs 900 kPa (Suppl Fig. 2).

Among the DEGs significantly upregulated on 900 kPa substrates across all ECMs, compared to those on 150 kPa substrates, we identified enriched pathways associated with ECM organization (*MMP14*, *COL5A1*, *COL6A1*), collagen formation (*LOX*, *COL8A1*, *ITGA6*) and Notch3 signaling that is involved in MSC differentiation (Fig. 6D). Conversely, the genes downregulated on 900 kPa, compared to 150 kPa, were enriched in pathways associated with focal adhesion, cell-substrate adhesion, and actin cytoskeleton gene sets (*NEXN*, *CNN3*, *LIMA1*) (Fig. 6E). Overall, the analysis of DEGs associated enrichment pathways suggests a strong stiffness-mediated regulation of MSC cellular processes, and that substrate stiffness differentially modulates ECM-related pathways.

**Fig. 7:**
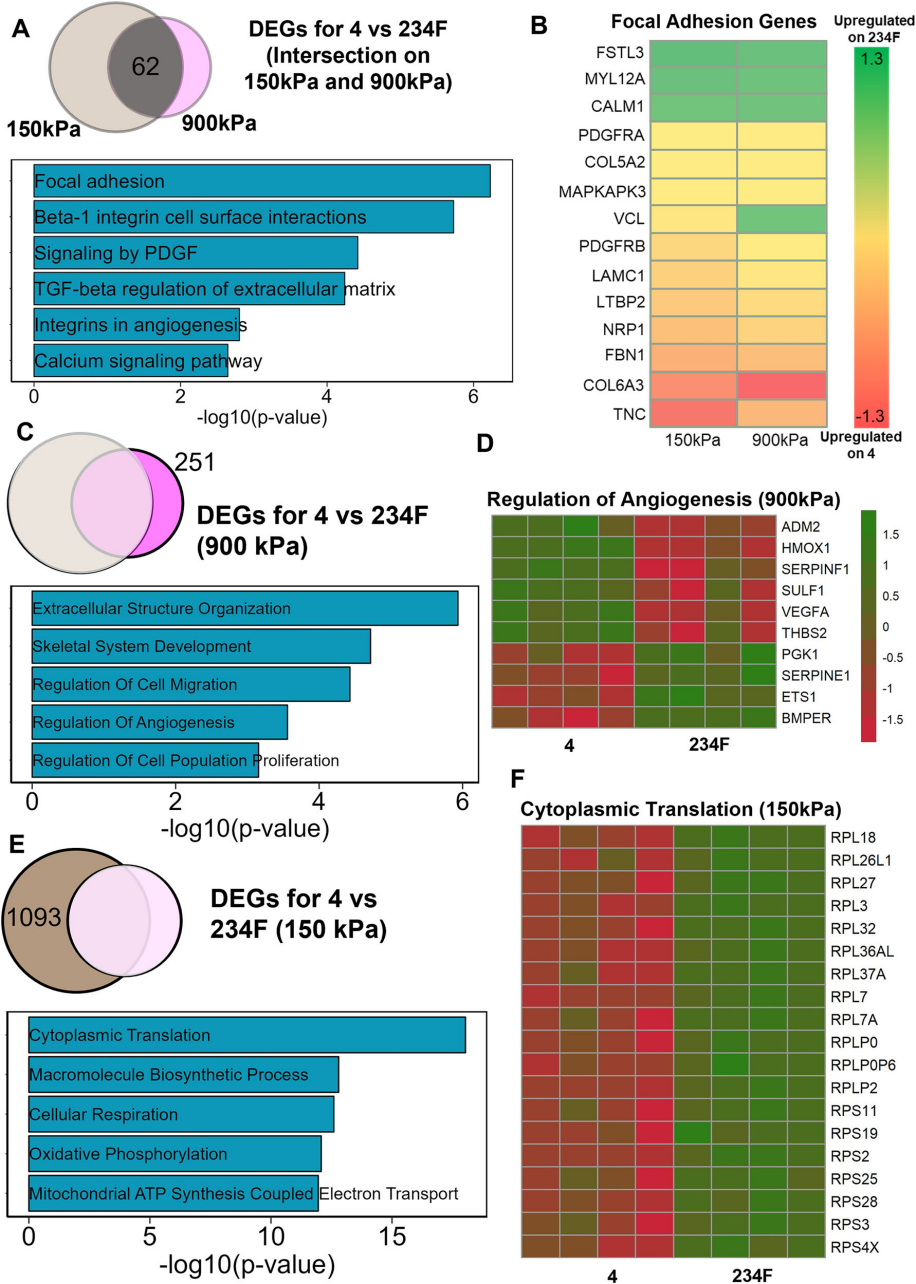
Transcriptional analysis of biochemical ECM effects on MSCs. **A** Venn Diagram and gene enrichment plot of common DEGs for comparison of 4 vs 234F at 150 kPa and 900 kPa. **B** Heatmap of focal adhesion related DEGs in the common 62 DEGs for comparison of 4 vs 234F at both 150 kPa and 900 kPa. **C** Venn Diagram and gene enrichment plot of DEGs for comparison of 4 vs 234F only on 900 kPa. **D** Heatmap of regulation of angiogenesis-related DEGs in the 251 DEGs for comparison of 4 vs 234F at 900 kPa. **E** Venn Diagram and gene enrichment plot of DEGs for comparison of 4 vs 234F only at 150 kPa **. F** Heatmap of cytoplasmic translation related DEGs in the 1093 DEGs for comparison of 4 vs 234F only at 150 kPa.

Having examined the stiffness effects, we then evaluated the effects of multi-component ECMs on MSCs by comparing the DEGs for the 4-factor combination, 234F, to that of the single-factor constituent, 4, on both 150 kPa and 900 kPa substrates. The Venn-diagram shows three sets of DEGs: (1) common DEGs for 4 vs 234F on both 150 kPa and 900 kPa (Fig. 7A); (2) DEGs for 4 vs 234F specifically on 900 kPa (Fig. 7C); and (3) DEGs for 4 vs 234F specifically on 150 kPa (Fig. 7E). Among the 62 DEGs found in comparing MSC transcriptome when cultured on 4 vs 234F on both stiffness (Fig. 7A), the common DEGs were enriched in pathways associated with focal adhesions, TGFβ regulation of ECM and integrin in angiogenesis (Fig. 7A, B). Among the focal adhesion genes, *FBN1* and *LAMC1* (ECM proteins) were upregulated on 4, whereas genes like *MYL12A* and *CALM1* (a calcium signaling protein) were upregulated on 234F (Fig. 7B). It was also observed that the ECM differences for the focal adhesion gene set were conserved at both stiffnesses, where the same set of genes were upregulated on 4 and 234F respectively (with the exception of *VCL*). Among the 251 DEGs when comparing MSCs cultured on 4 vs 234F on 900 kPa substrates, we found enrichment of these genes to skeletal system development, cell migration and angiogenesis (Fig. 7C, D). In the gene set “Regulation of Angiogenesis,” upregulation of *VEGFA* and *ADM2* (pro-angiogenic genes^30,31^) was found on 4 on 900 kPa substrates, whereas upregulation of *SERPINE1* (regulated expression of VEGF^32^, a pro-angiogenic factor) and *ETS1*^33^ (a transcription factor that regulate pro-angiogenic genes) was observed on 234F on 900 kPa stiffness. Conversely, among the 1093 DEGs on 150 kPa substrates, we found an enrichment in pathways like cytoplasmic translation, cellular respiration and oxidative phosphorylation (Fig. 7E, F). A significant upregulation of cytoplasmic translation genes was found on the 4-factor ECM combination 234F on 150 kPa, where more than 20 genes belonging to ribosomal protein subunit family (*RPL*s and *RPS*s) were upregulated on 234F compared to 4. Since ribosomal protein family subunits regulate protein translation in stem cell differentiation and embryonic development^34^, our results suggest more protein translation on 234F compared to 4 at 150 kPa. Overall, transcriptomic analysis revealed ECM-dependent pathways such as focal adhesions that are conserved on different stiffnesses, along with pathways such as angiogenesis and protein translation that are regulated by combinatorial ECMs on specific stiffness substrates.

### Clinical benchmark for comparing to MSC manufacturing in tissue chips

**Fig. 8:**
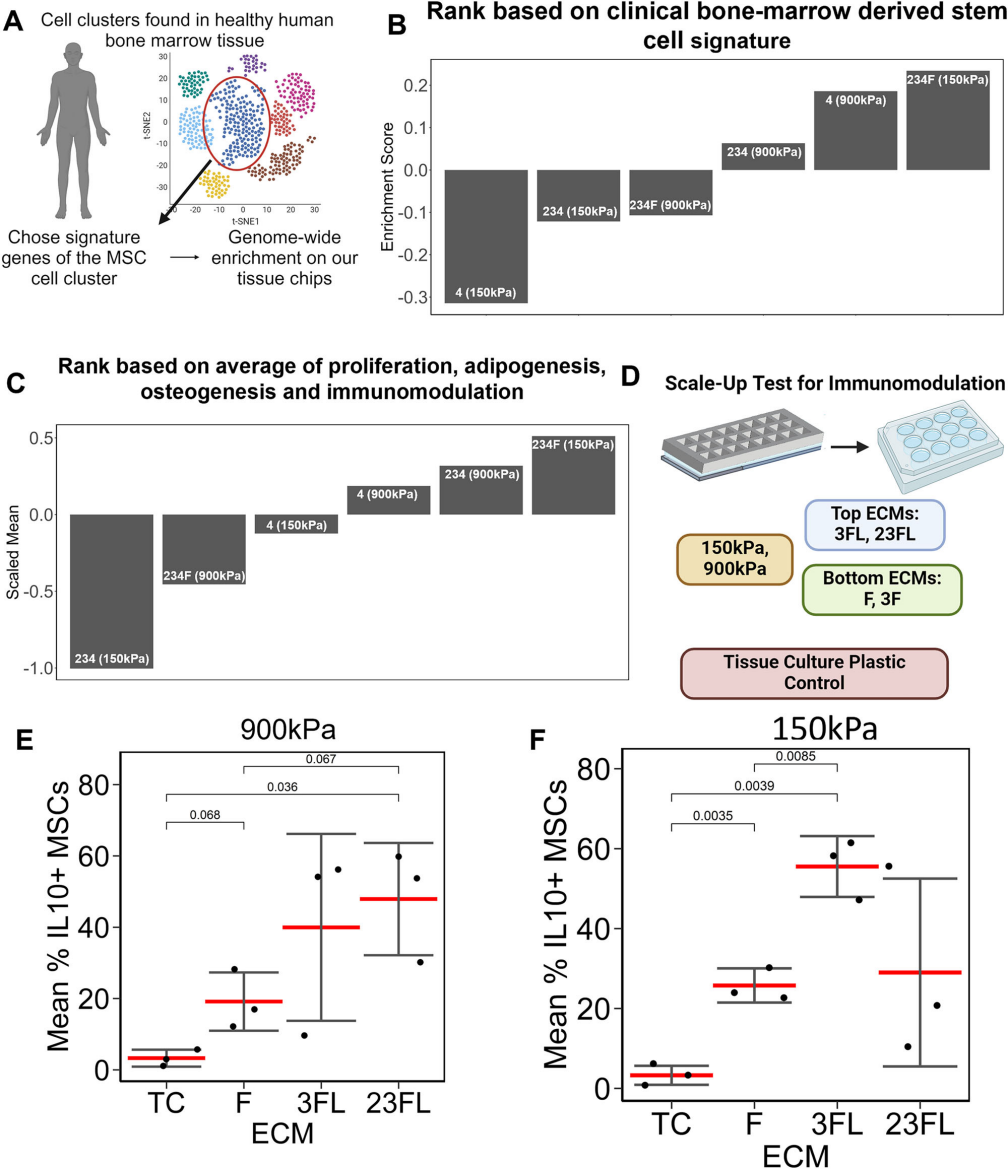
Clinical benchmark for tissue chips data. **A** Schematic of analysis pipeline for the analysis of clinical data. **B** Bar plot of ranked enrichment score derived from GSVA quantifying enrichment of clinical MSC gene signature in the tissue chips transcriptomics data. **C** Ranked averaged scaled mean of Oil Red O%+ MSCs, RUNX2%+ MSCs, Ki67%+ MSCs and IL-10%+ MSCs for the same ECM stiffness combinations as the transcriptomics data. **D** Schematic of a scale-up test for immunomodulation for MSC manufacturing. **E**, **F** Mean IL-10%+ MSCs as a function of ECM on 900 kPa and 150 kPa, respectively. Each dot indicates a biological replicate (*n* = 3). Created in BioRender. Huang, N. (2025) https://BioRender.com/j31b681.

Finally, we benchmarked the quality of MSCs cultured on tissue chips by comparing to published clinical datasets of MSCs obtained from freshly isolated healthy human bone marrow tissues^35^ (Fig. 8A). The single cell RNA Sequencing dataset identified various cell types within the bone marrow and the gene signatures associated with each cell type, including MSCs. Using the gene set defined for MSCs in the dataset, we performed Gene Set Variation Analysis (GSVA), which quantifies the relative enrichment of a gene set across multiple biological groups^36^. Through this analysis, we could quantify the enrichment of MSC gene signature that aligns best with the MSC gene set from the available clinical datasets using the transcriptomic data from the tissue chips. We then ranked the microenvironment conditions based on this enrichment. Based on this analysis, MSCs cultured on 234F at 150 kPa had the highest enrichment (Fig. 8B), suggesting that cells cultured on 234F at 150 kPa were most similar in transcriptome to freshly isolated MSCs (compared to MSCs cultured on other substrates). This result was consistent with rank ordering the scaled average metric of the same six microenvironmental conditions based on proliferation, adipogenesis, osteogenesis and immunomodulation (Fig. 8C). This comparison demonstrates the potential of tissue chips as a versatile tool for evaluating and benchmarking MSC phenotypes against clinical datasets, offering a framework for optimizing ECM conditions in manufacturing applications.

### Optimized ECM environments derived from tissue chip technology show improved immunomodulatory properties compared to conventional tissue culture polystyrene

Finally, we compared MSC manufacturing on tissue chips to tissue culture (TC) plastic as a reference control in a scalable manner. We quantified immunomodulation of MSCs cultured on 3FL and 23FL (top tier ECMs for IL-10+ cells) and F (bottom tier ECMs for IL-10%+ cells) and compared IL-10 expression against MSCs cultured on TC (Fig. 8D). These experiments were performed on 10-fold higher surface areas compared to tissue chips to demonstrate conservation of findings between tissue chips and higher scale culture of MSCs. Combinatorial ECMs on both 150 kPa and 900 kPa resulted in a significantly higher IL-10%+ MSCs, compared to TC. Specifically, 48% of MSCs cultured on 23FL respectively on 900 kPa were IL-10+ , compared to 3% IL-10+ MSCs on TC (Fig. 8E, *P* < 0.05). Similarly, 55% of MSCs cultured on 3FL at 150 kPa were IL-10+, compared to 3% IL-10+ MSCs on TC (Fig. 8F, *P* < 0.05). Furthermore, we also validated that top tier ECMs 3FL and 23FL had significantly higher IL-10%+ MSCs, compared bottom tier ECM, F. Specifically, 55% MSCs cultured on 3FL at 150 kPa were IL-10+, compared to 25% IL-10+ MSCs on F (Fig. 8F, *P* < 0.05) and 48% MSCs were IL-10+ on 23FL on 900 kPa, compared to 19% IL-10+ MSCs on F (Fig. 8E, close to significant *P* < 0.1). To further validate these findings, we performed a functional assay for testing MSC immunomodulation when cultured on the top ECM combination 3FL at 150 kPa and 900 kPa, compared to TC. The immunomodulation assay was performed by qualifying proliferation of activated T-cells co-cultured with MSCs on the different substrates. Our results show that MSCs cultured on 3FL at both 150 kPa and 900 kPa significantly reduced the % proliferating activated T-cells, compared to TC (Supplementary Fig. 3) This result confirms our finding of MSCs expressing higher IL-10 on 3FL environments, suggesting enhanced immunomodulation potential (Fig. 3C). Furthermore, we observed a trend (*P* < 0.1) of MSCs cultured on 900 kPa, resulting in lower percentage of proliferating activated T-cells, compared to MSCs cultured at 150 kPa. Overall, we demonstrated the scalability and improved immunomodulatory behavior of MSCs cultured on top tier ECM environments, compared to conventional TC dishes.

## Discussion

In this work, we developed a tissue chip platform to study multifactorial effects of substrate stiffness and ECM biochemical components on MSC behavior, transcriptional signature, and proteomic profile for manufacturing purposes. We specifically chose immunomodulation, adipogenic and osteogenic potential, and proliferation as measures of MSC behavior (Figs. 1–4). Mathematical modeling revealed non-intuitive insights on significant ECM combinations affecting these phenotypes. Furthermore, we performed proteomics analysis on multi-factorial ECM environments and found that the four-factor combination of 234 L resulted in a higher abundance among angiogenic and immunomodulatory factors, compared to one- and two-factor ECM compositions (Fig. 5). Transcriptomic analysis revealed mechanistic insights such as regulation of ECM on 900 kPa substrates and regulation of cell-substrate junction on 150 kPa substrates (Fig. 6). We further demonstrated that the regulation of angiogenesis and protein translation was driven by combinatorial ECMs on different stiffnesses (Fig. 7). Lastly, we evaluated our tissue-chips transcriptomics data using clinical dataset of healthy bone-marrow derived MSCs, which revealed 234F at 150 kPa substrates as most enriched, which corroborated with our in vitro findings (Fig. 8).

Currently, standard protocols for manufacturing cellular products involve culturing therapeutic cells on tissue culture plastic^37^, which are magnitudes higher in stiffness while lacking complex ECM milieus associated with where MSCs reside. This tissue chip platform may be used for developing customizable and optimal ECM environments for manufacturing MSCs from various donors or other cell types for cell therapy applications. Furthermore, it can be applied to the study of ECM effects on cells of diseased phenotypes. These studies also demonstrate that the optimal ECM and stiffness regime for MSCs in the context of manufacturing is dependent on the desired effect of the cellular therapy and/or desirable properties of the MSCs.

The tissue chip platform conveniently enables us to assess in parallel combinatorial ECM conditions, thereby revealing insights into how specific microenvironmental conditions affect different aspects of MSC behavior. For example, the highest proliferation was quantified on multifactorial ECM combinations, with the lowest proliferation found on a single factor ECM. Our proteomic data also revealed greater angiogenesis and immunomodulation on a 4-factor ECM combination, compared to single factor, 3-factor and 2-factor ECM combinations. Lastly, by comparing the transcriptomic profiles of MSCs cultured on tissue chips to a previously published transcriptome dataset of from freshly isolated bone marrow cells containing MSCs^35^, we identified that MSCs on 234F at 150 kPa exhibited the most similar gene expression profiles to in vivo human bone marrow stromal cells.

Among the main effects of ECM composition, in addition to combinatorial non-intuitive effects, we found 4 and L to have the most positive effects on adipogenesis, proliferation and immunomodulation. This is consistent with published literature studying single component ECMs on MSC behavior, in which 4 was reported to have a significant positive effect on differentiation and proliferation^38,39^. However, the L in these studies was not shown to have a significant effect^38,39^. This difference in finding could be due to masking of L’s effect in the combinatorial setting of our studies, which further underscore the importance of combinatorial environments. This study is the first of its kind to evaluate the effects of combinatorial ECMs and stiffnesses on MSC behavior and transcriptomics.

The differential effects of stiffness on proliferation and differentiation have been reported previously^13,14^. However, the range of stiffnesses evaluated in the literature has been relatively narrow (0.2 kPa–100 kPa). Notably, our study quantified stiffness effects from 150 kPa–900 kPa as a broader range than what has been studied in current literature. The lowest stiffness in our study is higher compared to most soft physiological soft tissues, but much softer than standard MSCs manufacturing substrates such as tissue culture plastic. The reason for choosing a range of stiffness that is higher than physiological stiffness is because the soft materials that mimic in vivo mechanical properties can be prone to technological limitations in manufacturing, owing to their fragility and faster degradation of physiologically relevant stiffnesses.

The improved immunomodulatory properties observed in MSCs cultured on top-tier ECM environments, compared to TC, highlights the practical benefits of our approach. Furthermore, some published works have shown that specific cell-cell interaction can modulate the effects of stiffness. For example, patterning of cells in a specific organization enhanced osteogenic differentiation of MSCs on stiff substrates^14^ and presentation of N-Cadherin dampened the downstream mechanosignaling pathways in MSCs on stiff substrates^40^. Our work demonstrated similar results where combinatorial ECMs enabled specific cellular pathways independent of the underlying stiffness.

This work expands the field by combining stiffness effects with multi-component ECMs, as well as by revealing new insights into MSC biology using transcriptomic and proteomic analyses specifically in the context of MSC manufacturing. Similar to our previous work, in which combinatorial ECMs enhanced myogenic differentiation of mouse myoblasts, especially in the presence of mechanical stretch^19^, we showed enhanced function of MSCs on combinatorial ECM, when compared to single factor ECMs. Furthermore, Hou et al. demonstrated enhanced endothelial differentiation of endothelial cells on combinatorial ECMs through integrin β3-mediated pathway^20^ and similar cellular mechanisms in the context of MSC manufacturing have been implicated in our study.

The scalable potential of these materials is high, as PDMS is widely available and easy to manufacture for cell culture applications. However, additional optimizations are needed to identify the minimum required ECM protein concentrations to reduce manufacturing costs. In addition to this point, the identified ECM combinations could be adapted to inform and optimize cell culture substrates made from materials already utilized in large-scale bioreactors, such as microcarriers or hydrogels. This adaptability could facilitate the integration of our findings into current manufacturing processes, enhancing scalability and production efficiency. Importantly, this tissue chip enables systematic optimization of multi-factorial ECM proteins and stiffness conditions for individual MSC donor lines while maintaining scalability for manufacturing. Future studies will expand this work by validating the platform across MSCs from multiple donors and tissue sources to enhance clinical applicability. We envision a translational direction in which cell therapies that require in vitro expansion will adopt the use of tissue chip platforms to identify customized optimal ECM microenvironments, moving away from conventional tissue culture polystyrene dishes for cell manufacturing.

In summary, our work systematically quantifies MSC behavior on tunable, multi-component ECM substrates using tissue chip technology, revealing previously unrecognized roles of ECM environments in MSC manufacturing. These insights pave the way for more efficient and scalable production of high-quality MSCs, with significant implications for regenerative medicine. By optimizing both the mechanical and biochemical aspects of the cell culture environment, we can enhance the therapeutic potential of MSCs, bringing us closer to their clinical adoption.

## Methods

### Cell culture

Bone marrow-derived MSCs (Lonza) were cultured in MSC growth media (MSCGM, Lonza) with 1% penicillin/streptomycin at 37 °C in a humidified atmosphere containing 5% CO_2_. Cells (passages 3–7) were sub-cultured when they reached 90% confluency.

### PDMS fabrication and mechanical characterization

Commercially available PDMS (Sylgard 184 and Sylgard 527) were mixed at different ratios including 1:1, 1:2, 1:4, 1:6. 1:9 (Sylgard 184:Sylgard 527). Each component was prepared according to the manufacturer’s instructions before mixing into the ratios then placed in 60 °C oven for 3 h to cure. The Young’s modulus of PDMS flat sheets (10 × 30 × 2 mm thickness) was measured on an Instron 5565 mechanical tester. Displacement and force measurements were taken at the rate of 1 cm/min until break and converted to stress and strain for Young’s modulus calculation in the elastic phase of the curve.

### ECM surface functionalization of PDMS tissue chip

Tissue chips were constructed with Proplate multi-well chamber (Grace BioLabs) with a flat sheet of PDMS derived from three different stiffnesses (150, 500, 900 kPa) and then secured with spring clamps. The surface of PDMS flat sheets were functionalized with ECMs including 2 (Adv BioMatrix), 3 (Adv BioMatrix), 4 (Southern Biotech), F (Sigma) and L (Life Technology), and their equimolar combinations thereof (1:1, 1:1:1, etc). This was done using a polydopamine crosslinker^41^. Briefly, sterilized assembled tissue chips are incubated with freshly prepared L-dopamine (0.01% in Tris-HCl buffer, 10 mM, pH 8.5, Sigma) for 1 h at room temperature. Tissue chips were washed with PBS and then incubated with ECM solutions reconstituted in PBS at a concentration of 2.7 µg/cm^2^ for 1 h at room temperature. After washing again with PBS, the chips were used for in vitro studies.

### Proliferation

MSC were seeded at 1 × 10^4^/cm^2^ for each well of the tissue chip and allowed to attach overnight before changing to fresh growth media and allowed to grow for 48 h. Cells were fixed with 4% paraformaldehyde for 10 min at room temperature. Cells were probed for anti-Ki67 antibody (Dako) as a marker for proliferation, along with phalloidin (Fisher Scientific) for F-actin assembly, and Hoechst33342 (Fisher Scientific) for total nuclei. In addition, the population growth rate was tracked in a subset of ECM conditions comprising the highest or lowest ranked ECMs on 500 kPa using brightfield images that were taken after 24 h. The total cell numbers were counted.

### Immunomodulation

MSC were seeded at 2 × 10^4^/cm^2^ for each well and allowed to attach overnight before changing to fresh growth media with IFN-γ (25 ng/mL) for 2 h. Cells were fixed with 4% paraformaldehyde for 10 min at room temperature. Cells were probed for anti-IL-10 antibody (Santa Cruz Biotech) along with phalloidin (Fisher Scientific) for F-actin assembly, and Hoechst33342 for total nuclei. The same protocol was followed for tissue chips and scale-up experiment in 12-well TC plate.

### Adipogenic differentiation

MSC were seeded at 2 × 10^4^ /cm^2^ for each well and allowed to grow to confluency before changing to adipogenic differentiation media (Lonza). After three cycles of adipogenic media inductions, cells were fixed with 4% paraformaldehyde for 15 min at room temperature. Cells were then stained with Oil Red O (Sigma) to visualize lipid droplets and counter stained with Hoechst33342 for nuclei.

### Osteogenic differentiation

MSCs were seeded at 2 × 10^4^ /cm^2^ for each well and allowed to grow to confluency before changing to osteogenic differentiation media (Lonza). The differentiation induction was performed for 21 days with a media change every 4 days. At the end cells were fixed with 4% paraformaldehyde for 15 min followed by immunostaining for RUNX2 primary antibody (ThermoFisher). The cells were also stained with Hoechst33342 for nuclei visualization.

### Image analysis

Images were captured using 10× objectives on inverted microscope system (BZ-X710, Keyence). For Ki67 analysis of cell proliferation, Ki67+ cells were expressed as a percentage of total nuclei count (*n* = 3). IL-10+ cells were represented as number of cells with IL-10 staining, normalized to the total nuclei count (*n* = 3). Adipogenic differentiation was assessed by counting the number of Oil Red O positively stained cells as a percentage of total nuclei count (*n* = 3). RUNX2+ cells were calculated by defining a threshold for RUNX2 intensity and calculating the RUNX2%+ cells for every image (*n* = 3).

### Proteomic analysis

The secretome of the MSCs after adipogenic differentiation was analyzed (*n* = 3 per ECM composition). After completing the adipogenic differentiation procedure, the media was changed to serum free DMEM and allowed to incubate for 48 h. The conditioned media was collected and analyzed using commercial cytokine arrays for angiogenesis and immunomodulation markers (RayBiotech). The plate reader fluorescent values obtained from the cytokine array was normalized to the cell numbers for each condition. Surrogate variable analysis was done using RUVseq package in R, and removeBatchEffect function was used to remove the surrogate variables found. Differences between the secretome were identified using lmFit function of package limma in R. Specific comparisons (stiffness/ECM) were statistically tested in the model using contrasts.fit function. An adjusted *P* value (Padj) and fold change were calculated for each region and comparison.

### Quantitative PCR (qPCR)

Reverse transcription on isolated RNA sample was performed using the Superscript II reverse transcriptase (Invitrogen). The cDNA was synthesized according to Invitrogen’s protocol on First Strand cDNA synthesis. Primers used for Taqman qPCR consisted of ANGPTL4, MYOCD and GAPDH (all from Applied Biosystems, Foster City, CA). The qPCR was performed on a 7900 real-time PCR system (Applied Biosystems) for 40 cycles. The data were analyzed and processed by the ΔΔCt method, normalized to GAPDH housekeeping gene, and then quantified as normalized relative fold changes with respect to 4 ECM condition on 900 kPa (*n* ≥ 3).

### RNA sequencing analysis

Short term gene changes were assessed in MSCs seeded on tissue chips on 4, 234 and 234F ECM combinations at 150 kPa and 900 kPa, following the proliferation protocol. After 48 h in growth media, cells were lysed and RNA was extracted using GeneJET RNA isolation kit (Fisher Scientific) followed by RNA sequencing by Novogene Corporation (*n* = 4). In addition, RNA Sequencing analysis was performed using publicly available datasets derived from freshly isolated human bone marrow cells containing MSCs^35^ (GSE190965). These clinical datasets were derived from Skåne University Hospital with informed consent and the use of human samples was approved by the Regional Ethics Review Board in Lund, Sweden. Analysis of this dataset was performed in accordance with the Declaration of Helsinki.

CASAVA base recognition (Base Calling) was used to convert original image data files from Illumina sequencing platform to sequenced reads in FASTQ format. A series filtration criteria was applied based on uncertain nucleotides constituting more than 10% of either read (*n* > 10%) and with low-quality nucleotides (base quality less than 5) constituting more than 50 per cent of the read. Next, alignment of raw reads was performed to reference genome using HISAT2. To estimate gene expression levels, the fragments per kilobase of transcript sequence per million base pairs sequenced (FPKM) was quantified for each aligned read. This accounted for both sequencing depth and gene length on counting of fragments.

For expression analysis, the FPKM counts were normalized and log transformed using edgeR function in R. This was followed by linear modeling of gene expression using lmFIt function in limma package in R to obtain Differential Expressed (DEGs). Specific comparisons (stiffness/ECM) were statistically tested in the model using contrasts.fit function where Adjusted P value (Padj) and fold change was calculated for each region and comparison. Specific differential gene expression lists were made for specific comparisons to enable enrichment analysis on EnrichR website. For gene set variation analysis (GSVA) analysis for clinical datasets, R package gsva was utilized. Specifically, marker genes for human bone-marrow derived MSCs from human clinical samples were used as the gene set for getting the enrichment score and setting the clinical benchmark. Finally, ggplot and pheatmap packages in R were used to create bar plots and heatmaps.

### Statistics

All data is shown as mean ± standard deviation. For RNA sequencing and proteomics, a linear model was computed for the gene expression as function of the ECM and mechanical stimulus (three biological replicate each). The differences between each treatment group were calculated using established methods in the contrasts.fit function in limma package R. The statistics for the differential gene expression is calculated using eBayes functions in which analysis of variance (ANOVA) with Tukey’s post hoc test was performed for all comparisons. Statistical significance was accepted at Padj < 0.05. All other statistical analyses were performed by analysis of variance (ANOVA) with Tukey post hoc test. Linear regression models were adopted to estimate coefficients of main effects for each variable (adipogenesis, osteogenesis, immunomodulation and proliferation), which were calculated using lm function in R. The full-factorial ANOVA, including all ECM combinations, were performed through the generalized linear models (GLM) in SAS (version 9.4, SAS Institute Inc. NC, USA). Statistical significance was accepted at *P* < 0.05.

## Supplementary information

Supplementary Information

## Data Availability

The RNA Sequencing datasets derived from MSCs in this study are available from the Gene Expression Omnibus at GSE288678. RNA Sequencing analysis was also performed using publicly available datasets derived from freshly isolated human bone marrow cells containing MSCs^35^ (GSE190965). All other data will be made available upon reasonable request.

## Code availability

In this work, only public software and custom code were used to generate or process datasets. The software version and websites were described in the “Methods” section.

## References

[1] Soliman, H. et al. Multipotent stromal cells: One name, multiple identities. *Cell Stem Cell* **28**, 1690–1707, 10.1016/j.stem.2021.09.001 (2021). 34624231 10.1016/j.stem.2021.09.001

[2] Phinney, D. G. & Prockop, D. J. Concise review: Mesenchymal stem/multipotent stromal cells: The state of transdifferentiation and modes of tissue repair—current views. *Stem Cells* **25**, 2896–2902, 10.1634/stemcells.2007-0637 (2007). 17901396 10.1634/stemcells.2007-0637

[3] Dzobo, K. Recent trends in multipotent human mesenchymal stem/stromal cells: learning from history and advancing clinical applications. *OMICS* **25**, 342–357, 10.1089/omi.2021.0049 (2021). 34115524 10.1089/omi.2021.0049

[4] Galderisi, U., Peluso, G. & Di Bernardo, G. Clinical trials based on mesenchymal stromal cells are exponentially increasing: Where are we in recent years?. *Stem Cell Rev. Rep.* **18**, 23–36, 10.1007/s12015-021-10231-w (2022). 34398443 10.1007/s12015-021-10231-w PMC8365566

[5] Zanetti, C. & Krause, D. S. “Caught in the net”: the extracellular matrix of the bone marrow in normal hematopoiesis and leukemia. *Exp. Hematol.* **89**, 13–25, 10.1016/j.exphem.2020.07.010 (2020). 32755619 10.1016/j.exphem.2020.07.010

[6] Lin, D., Chun, T. H. & Kang, L. Adipose extracellular matrix remodelling in obesity and insulin resistance. *Biochem Pharm.* **119**, 8–16, 10.1016/j.bcp.2016.05.005 (2016). 27179976 10.1016/j.bcp.2016.05.005 PMC5061598

[7] Mariman, E. C. M. & Wang, P. Adipocyte extracellular matrix composition, dynamics and role in obesity. *Cell. Mol. Life Sci.* **67**, 1277–1292, 10.1007/s00018-010-0263-4 (2010). 20107860 10.1007/s00018-010-0263-4 PMC2839497

[8] Ruiz-Ojeda, F. J., Méndez-Gutiérrez, A., Aguilera, C. M. & Plaza-Díaz, J. Extracellular matrix remodeling of adipose tissue in obesity and metabolic diseases. *Int. J. Mol. Sci.* **20**, 4888, 10.3390/ijms20194888 (2019). 31581657 10.3390/ijms20194888 PMC6801592

[9] Lee-Thedieck, C., Schertl, P. & Klein, G. The extracellular matrix of hematopoietic stem cell niches. *Adv. Drug Deliv. Rev.* **181**, 114069, 10.1016/j.addr.2021.114069 (2022). 34838648 10.1016/j.addr.2021.114069 PMC8860232

[10] Woods K., Guezguez B. Dynamic changes of the bone marrow niche: Mesenchymal stromal cells and their progeny during aging and leukemia. *Front Cell Dev Biol.* **9**. 10.3389/fcell.2021.714716. (2021) 10.3389/fcell.2021.714716 PMC8383146 34447754

[11] Crisan, M. et al. A perivascular origin for mesenchymal stem cells in multiple human organs. *Cell Stem Cell* **3**, 301–313, 10.1016/j.stem.2008.07.003 (2008). 18786417 10.1016/j.stem.2008.07.003

[12] Tse, J. R. & Engler, A. J. Stiffness gradients mimicking in vivo tissue variation regulate mesenchymal stem cell fate. *PLoS One* **6**, e15978, 10.1371/journal.pone.0015978 (2011). 21246050 10.1371/journal.pone.0015978 PMC3016411

[13] Vilar, A. et al. Substrate mechanical properties bias MSC paracrine activity and therapeutic potential. *Acta Biomater.* **168**, 144–158, 10.1016/j.actbio.2023.06.041 (2023). 37422008 10.1016/j.actbio.2023.06.041

[14] Mao, A. S., Shin, J. W. & Mooney, D. J. Effects of substrate stiffness and cell-cell contact on mesenchymal stem cell differentiation. *Biomaterials* **98**, 184–191, 10.1016/j.biomaterials.2016.05.004 (2016). 27203745 10.1016/j.biomaterials.2016.05.004 PMC4906313

[15] Alakpa, E. V. et al. Tunable supramolecular hydrogels for selection of lineage-guiding metabolites in stem cell cultures. *Chem* **1**, 298–319, 10.1016/j.chempr.2016.07.001 (2016).

[16] Kureel S. K. et al. Soft substrate maintains proliferative and adipogenic differentiation potential of human mesenchymal stem cells on long-term expansion by delaying senescence. *Biol. Open*; **8**. 10.1242/bio.039453 (2019). 10.1242/bio.039453 PMC6503999 31023646

[17] Wade, R. J. & Burdick, J. A. Engineering ECM signals into biomaterials. *Mater. Today* **15**, 454–459, 10.1016/S1369-7021(12)70197-9 (2012).

[18] Lukashev, M. & Werb, Z. ECM signalling: Orchestrating cell behaviour and misbehaviour. *Trends Cell Biol.* **8**, 437–441, 10.1016/S0962-8924(98)01362-2 (1998). 9854310 10.1016/s0962-8924(98)01362-2

[19] Chan, A. H. P. et al. Combinatorial extracellular matrix cues with mechanical strain induce differential effects on myogenesis in vitro. *Biomater. Sci.* **11**, 5893–5907, 10.1039/d3bm00448a (2023). 37477446 10.1039/d3bm00448a PMC10443049

[20] Hou, L. et al. Combinatorial extracellular matrix microenvironments for probing endothelial differentiation of human pluripotent stem cells. *Sci. Rep.* **7**, 6551. 10.1038/s41598-017-06986-3 (2017). 28747756 10.1038/s41598-017-06986-3 PMC5529516

[21] Brougham-Cook, A. et al. High throughput interrogation of human liver stellate cells reveals microenvironmental regulation of phenotype. *Acta Biomater.* **138**, 240–253, 10.1016/j.actbio.2021.11.015 (2022). 34800715 10.1016/j.actbio.2021.11.015 PMC8738161

[22] Ryoo H., Giovanni R., Kimmel H., Jain I., Underhill G. H. Combinatorial Microgels for 3D ECM Screening and Heterogeneous Microenvironmental Culture of Primary Human Hepatic Stellate Cells. *Adv. Sci.* **11**. 10.1002/advs.202303128 (2024). 10.1002/advs.202303128 PMC11022709 38348560

[23] Kaylan, K. B. et al. Spatial patterning of liver progenitor cell differentiation mediated by cellular contractility and Notch signaling. *Elife*; **7**. 10.7554/eLife.38536 (2018). 10.7554/eLife.38536 PMC6342520 30589410

[24] Ireland, R. G. et al. Combinatorial extracellular matrix microarray identifies novel bioengineered substrates for xeno-free culture of human pluripotent stem cells. *Biomaterials* **248**, 120017, 10.1016/j.biomaterials.2020.120017 (2020). 32283392 10.1016/j.biomaterials.2020.120017

[25] Zuur A. F., Ieno E. N., Walker N. J., Saveliev A. A., Smith G. M. GLM and GAM for Absence–presence and proportional data, 245-259 10.1007/978-0-387-87458-6_10 (2009).

[26] Izzi, V. et al. An extracellular matrix signature in leukemia precursor cells and acute myeloid leukemia. *Haematologica* **102**, 1807–1809, 10.3324/haematol.2017.167304 (2017). 28411251 10.3324/haematol.2017.167304 PMC5566047

[27] Aggarwal, S. & Pittenger, M. F. Human mesenchymal stem cells modulate allogeneic immune cell responses. *Blood* **105**, 1815–1822, 10.1182/blood-2004-04-1559 (2005). 15494428 10.1182/blood-2004-04-1559

[28] Groux, H., Bigler, M., de Vries, J. E. & Roncarolo, M.-G. Inhibitory and stimulatory effects of IL-10 on human CD8+ T cells. *J. Immunol.* **160**, 3188–3193, 10.4049/jimmunol.160.7.3188 (1998). 9531274

[29] Zhang, C. et al. Il-10 mrna engineered mscs demonstrate enhanced anti-inflammation in an acute gvhd model. *Cells* **10**, 3101, 10.3390/cells10113101 (2021). 34831324 10.3390/cells10113101 PMC8621791

[30] Holmes, D. I. & Zachary, I. The vascular endothelial growth factor (VEGF) family: Angiogenic factors in health and disease. *Genome Biol.* **6**, 209. 10.1186/gb-2005-6-2-209 (2005). 15693956 10.1186/gb-2005-6-2-209 PMC551528

[31] Wang, F. et al. Accelerated bone regeneration by adrenomedullin 2 through improving the coupling of osteogenesis and angiogenesis via β-catenin signaling. *Front Cell Dev. Biol.* **9**, 649277, 10.3389/fcell.2021.649277 (2021). 33937244 10.3389/fcell.2021.649277 PMC8079771

[32] Balsara, R. D. & Ploplis, V. A. Plasminogen activator inhibitor-1: The double-edged sword in apoptosis. *Thromb. Haemost.* **100**, 1029–1036, 10.1160/TH08-07-0427 (2008). 19132226 PMC3674867

[33] Iwasaka, C., Tanaka, K., Abe, M. & Sato, Y. Ets-1 regulates angiogenesis by inducing the expression of urokinase-type plasminogen activator and matrix metalloproteinase-1 and the migration of vascular endothelial cells. *J. Cell Physiol*. **169**, 522–531 (1996). 10.1002/(SICI)1097-4652(199612)169:33.0.CO;2-7. 10.1002/(SICI)1097-4652(199612)169:3<522::AID-JCP12>3.0.CO;2-7 8952701

[34] Li D., Wang J. Ribosome heterogeneity in stem cells and development. *J. Cell Biol.* **219**. 10.1083/JCB.202001108 (2020). 10.1083/jcb.202001108 PMC7265316 32330234

[35] Li H. et al. Identification of phenotypically, functionally, and anatomically distinct stromal niche populations in human bone marrow based on single-cell RNA sequencing. *Elife* **12**. 10.7554/elife.81656 (2023). 10.7554/eLife.81656 PMC10097421 36876630

[36] Hänzelmann, S., Castelo, R. & Guinney, J. GSVA: Gene set variation analysis for microarray and RNA-Seq data. *BMC Bioinforma.* **14**, 7. 10.1186/1471-2105-14-7 (2013). 10.1186/1471-2105-14-7 PMC3618321 23323831

[37] Phinney, D. G. & Galipeau, J. Manufacturing mesenchymal stromal cells for clinical applications: A survey of Good Manufacturing Practices at U.S. academic centers. *Cytotherapy* **21**, 782–792, 10.1016/j.jcyt.2019.04.003 (2019). 31182333 10.1016/j.jcyt.2019.04.003

[38] Clements, L. E., Garvican, E. R., Dudhia, J. & Smith, R. K. W. Modulation of mesenchymal stem cell genotype and phenotype by extracellular matrix proteins. *Connect Tissue Res* **57**, 443–453, 10.1080/03008207.2016.1215442 (2016). 27448620 10.1080/03008207.2016.1215442

[39] Ode, A. et al. Toward biomimetic materials in bone regeneration: Functional behavior of mesenchymal stem cells on a broad spectrum of extracellular matrix components. *J. Biomed. Mater. Res A* **95**, 1114–1124, 10.1002/jbm.a.32909 (2010). 20878902 10.1002/jbm.a.32909

[40] Cosgrove, B. D. et al. N-cadherin adhesive interactions modulate matrix mechanosensing and fate commitment of mesenchymal stem cells. *Nat. Mater.* **15**, 1297–1306, 10.1038/nmat4725 (2016). 27525568 10.1038/nmat4725 PMC5121068

[41] Chuah, Y. J. et al. Simple surface engineering of polydimethylsiloxane with polydopamine for stabilized mesenchymal stem cell adhesion and multipotency. *Sci. Rep.* **5**, 18162. 10.1038/srep18162 (2015). 26647719 10.1038/srep18162 PMC4673458

