# Supplementary material for: Combinatorial extracellular matrix tissue chips for optimizing mesenchymal stromal cell microenvironment and manufacturing
Source: NPJ Regen Med. 2025 Apr 22;10:21. doi: 10.1038/s41536-025-00408-z (PMC12015357; doi:10.1038/s41536-025-00408-z)
Supplement: Supplementary file 1 — Supplementary Information [file 41536_2025_408_MOESM1_ESM.pdf]

Supplementary Figures

Combinatorial Extracellular Matrix Tissue Chips for Optimizing Mesenchymal Stromal Cell Microenvironment and Manufacturing

Ishita Jain[1,2#], Alex H.P. Chan[1,2#], Guang Yang[1,3], Hao He[1], Johnny Lam[4], Kyung Sung[4], Ngan F. Huang[1,2,5,6*]

*To whom correspondence should be addressed:
Dr. Ngan F. Huang, PhD
Associate Professor Department of Cardiothoracic Surgery
Stanford University
300 Pasteur Drive, MC 5407
Stanford, CA 94305-5407
Tel: 650-849-0559
Email: ngantina@stanford.edu
ORCID Identifier: 0000-0003-2298-6790

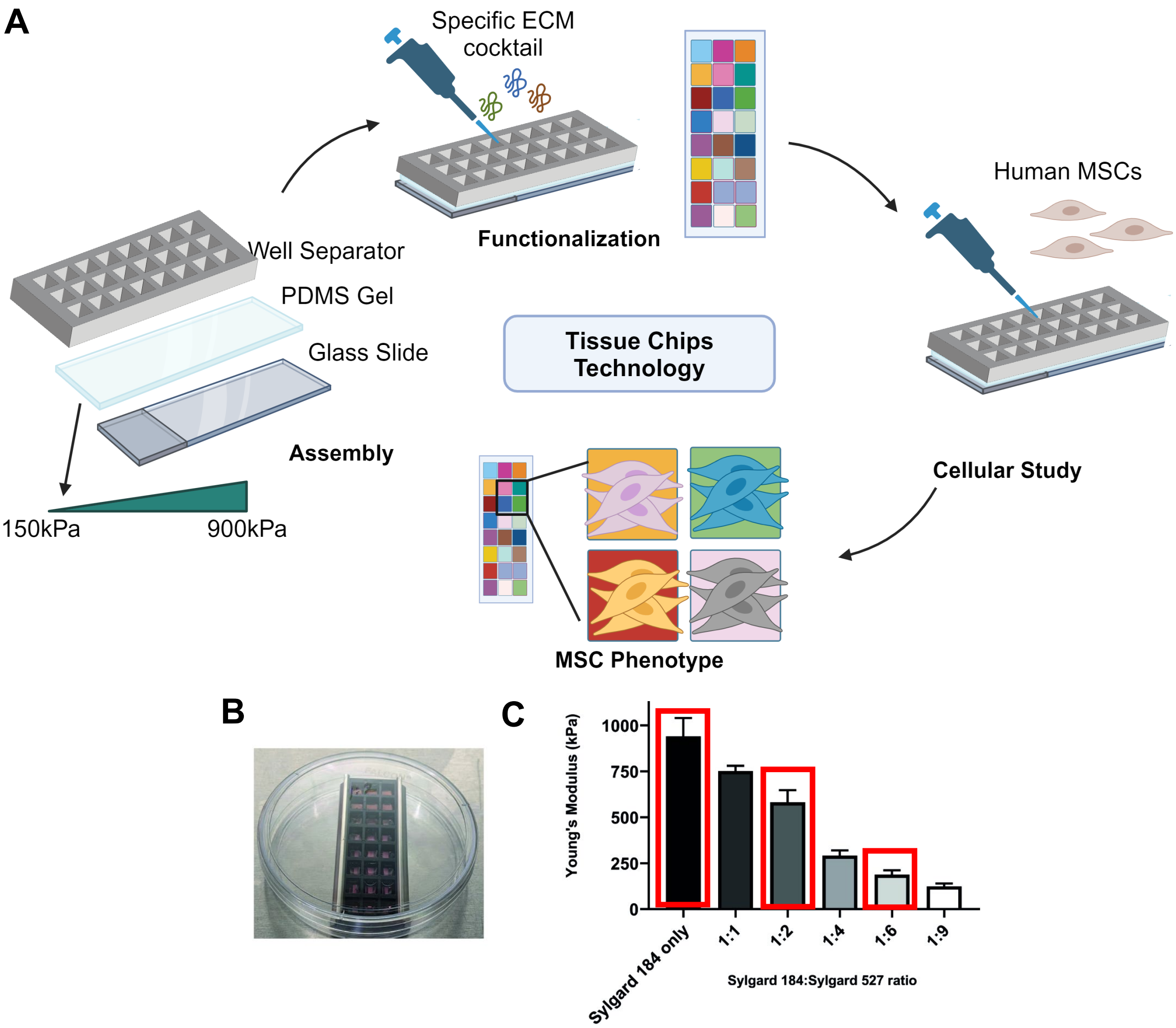

Supplementary Figure 1: Summary and Development of tissue chips technology. A. Schematic of tissue chips technology design and process of evaluating MSCs phenotype as a function of extracellular matrix components and stiffness. B) Representative image of assembled tissue chips. **B.** Young's modulus (kPa) of different ratios of PDMS polymers Sylgard 184 and Sylgard 527. The bars with the red boxes were chosen for the all the experiments in this paper. Created in BioRender. Huang, N. (2025) https://BioRender.com/b97k029.

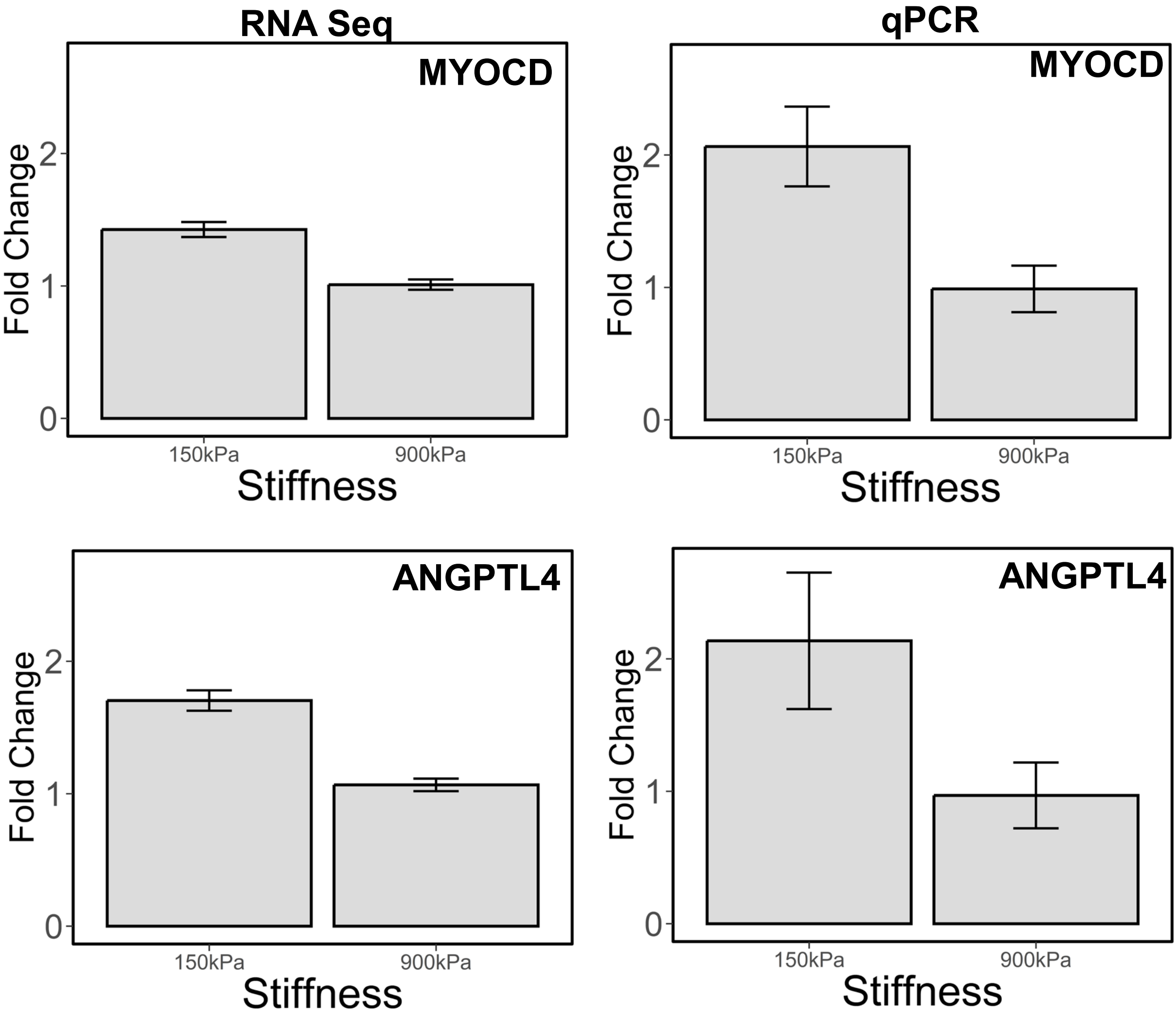

Supplementary Figure 2: Validation of findings from RNAseq data using PCR. Fold change with respect to ECM condition 4 on 900 kPa calculated for both RNAseq and PCR. Housekeeping gene for PCR was GAPDH (n≥3).

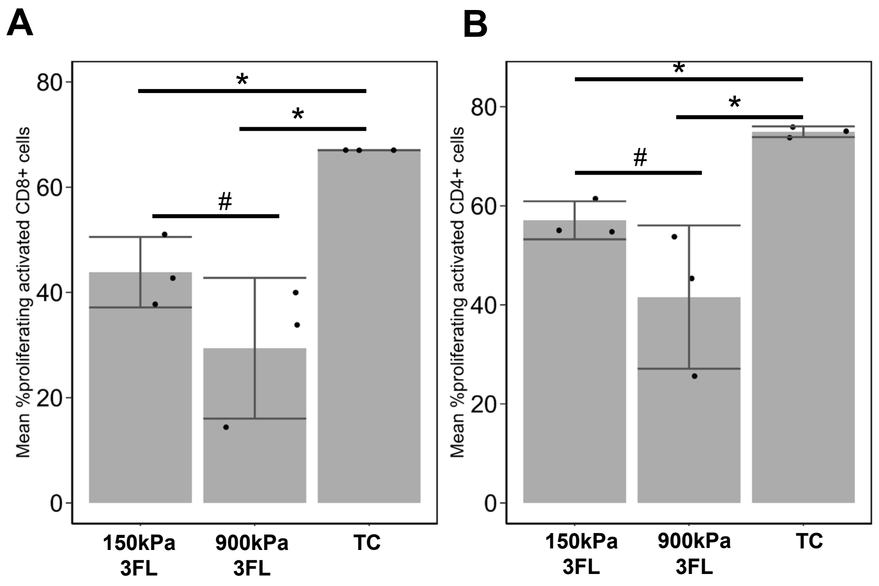

Supplementary Figure 3: Validation of findings from immunomodulation data using a function PBMC (Peripheral Blood Mononuclear Cells) assay. A-B. Percentage of proliferating activated CD8+ and CD4+ T cells in PBMC activated using Dynabeads and co-cultured with MSCs on 3FL (150kPa), 3FL (900kPa) and tissue culture polystyrene (TC). * represents P<0.05, # represents P<0.1 (n=3).
